# Supplementary figures and images for: Rab11a mediates cell-cell spread and reassortment of influenza A virus genomes via tunneling nanotubes
Source: PLoS Pathog. 2021 Sep 2;17(9):e1009321. doi: 10.1371/journal.ppat.1009321 (PMC8443049; doi:10.1371/journal.ppat.1009321)

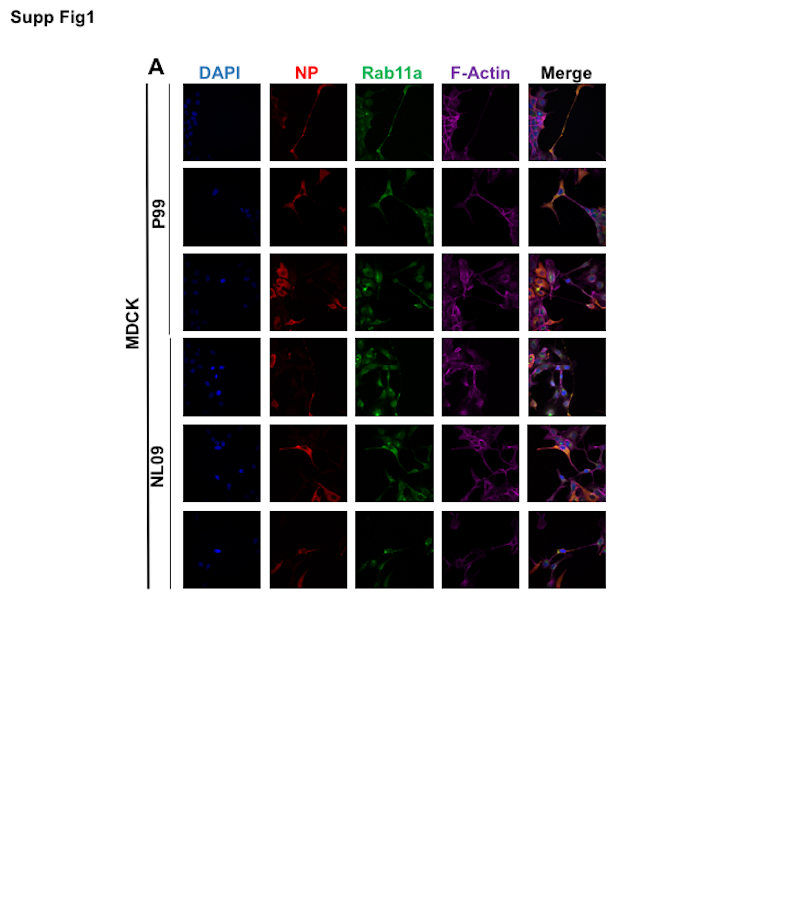

Supplement: S1 Fig — MDCK cells were mock-infected or infected with NL09 or P99 viruses. Cells were stained for DAPI [blue], NP [red], Rab11a [green] and F-Actin [pink]. Scale bar is 20μm for all images. (TIF) [file ppat.1009321.s001.tif]

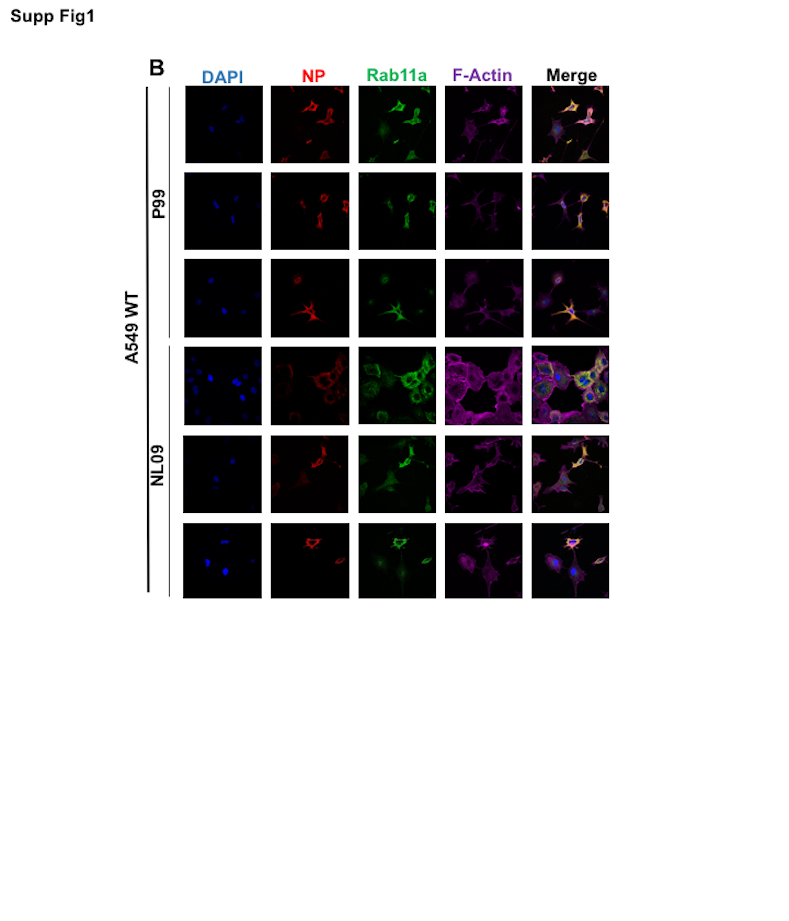

Supplement: S2 Fig — A549 WT cells were mock-infected or infected with NL09 or P99 viruses. Cells were stained for DAPI [blue], NP [red], Rab11a [green] and F-Actin [pink]. Scale bar is 20μm for all images. (TIF) [file ppat.1009321.s002.tif]

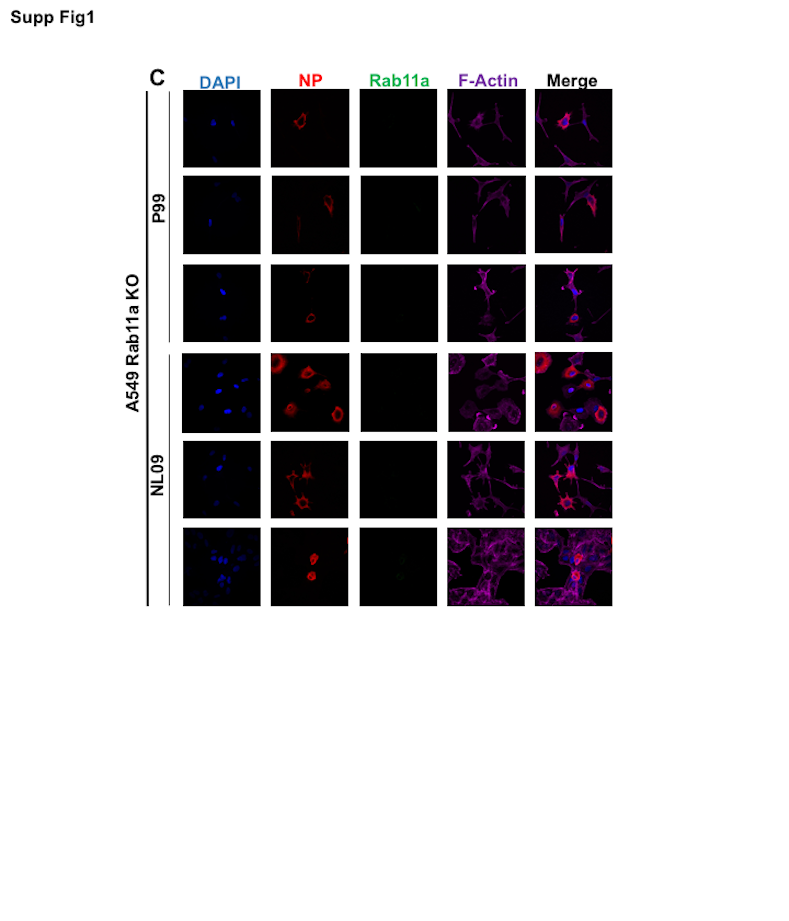

Supplement: S3 Fig — A549 Rab11a KO cells were mock-infected or infected with NL09 or P99 viruses. Cells were stained for DAPI [blue], NP [red], Rab11a [green] and F-Actin [pink]. Scale bar is 20μm for all images. (TIF) [file ppat.1009321.s003.tif]
