# Supplementary material for: Rab11a mediates cell-cell spread and reassortment of influenza A virus genomes via tunneling nanotubes
Source: PLoS Pathog. 2021 Sep 2;17(9):e1009321. doi: 10.1371/journal.ppat.1009321 (PMC8443049; doi:10.1371/journal.ppat.1009321)
Supplement: Attachment — Submitted filename: Responses to Reviewers.docx [file ppat.1009321.s005.docx]

Dear Dr. Lopez,

We are happy to submit a revised version of our manuscript entitled “Rab11a mediates cell-cell spread and reassortment of influenza A virus genomes via tunneling nanotubes." (PPATHOGENS-D-21-00131). We have reproduced the reviewers' comments on the following pages and detailed our responses underneath in blue font. The modifications made include the addition of new experimental data and clarifications within the text. The reviewers provided insightful comments and we would like to thank them for improving the quality of this work.

Sincerely,

Ketaki Ganti and Anice Lowen

**Part I –Major Concerns**

Reviewer #1: Additional Experiments

1) The authors comment on colocalization of Rab11a with NP inside TNTs in Fig 1, yet this is very difficult to discern from the images provided. NP appears to fill the entire cytosol- Higher magnification / better resolution of the TNTs in these images would be helpful- Also, colocalization analysis would strengthen their argument (e.g. does 100% of NP within TNTs colocalize with Rab11a?)

We thank the reviewer for their comments. We have included high resolution STED images to show NP-Rab11a co-localization within TNTs in Figure 2A. We have also included statistical analyses to show co-localization efficiency of Rab11a and NP within TNTs in Figure 2B. These results are described in the modified text at lines 112-123, as follows:

“We used super resolution Stimulated Emission Depletion (STED) microscopy to analyze the association of Rab11a and vRNPs within TNTs in more detail. STED microscopy overcomes the diffraction resolution limit of confocal microscopy and allows for imaging with up to 30 nm resolution (24,25). A549 WT or Rab11a KO cells were infected with NL09 viruses and stained for NP, Rab11a and F-Actin. Using 3 color STED imaging of TNTs, we observed punctate Rab11a and NP staining in close proximity within the TNTs in WT cells. In the case of the Rab11a KO cells, the NP staining was relatively diffuse in the cytoplasm and could not be observed within the TNTs (Figure 2A). Co-localization of Rab11a and NP within TNTs was analyzed quantitatively for both WT and KO cells. The observed colocalization co-efficient of 0.9 in the WT cells indicates that the majority of the NP puncta within TNTs coincide with Rab11a puncta (Figure 2B). These data indicate that Rab11a and NP are indeed interacting with each other within TNTs and are likely trafficked as a complex.”

Note that one of the images in the main figure for the KO cells appears to be duplicated in the supplemental figure (S1C)

We thank the reviewer for catching this error and have made the correction to Supplemental Figure 1C.

2) As no characterization of the KO cells is provided beyond the lack of Rab11a staining in IF images, a rescue experiment would provide confirmation that the phenotype (loss of foci formation) is indeed specific for Rab11a.

We have included a reference for our recently published paper at line 106 (Han et al. 2021) where the complete characterization of the KO cells has been described in detail. The paper shows validation of the KO cells by western blot and transferrin uptake assay. Although the reviewers make a great point about the rescue of the phenotype to confirm the role of Rab11a in our foci formation experiment, technical limitations, namely poor transfection efficiency, is a barrier in generating these data.

3) The authors suggest that the production of infectious virus in Fig 4C could be due to the action of Rab11a from the HA- expressing recipient cells. To support this, they coculture A549 cells expressing Rab11a-mCherry with IAV-infected Rab11a KO cells and note that they “observe colocalization of Rab11a and NP in the Rab KO cells, indicating the transfer of mCherry Rab11a to the infected cells.” How do we know that this is Rab11a-mCherry in KO cells and not NP transfer to Rab-mCherry expressing cells? Overall these data are not sufficient to support the conclusion that TNTs allow bidirectional movement and that Rab11a from uninfected cells can essentially grab RNPs from their neighbours.

We agree with the reviewer that the data originally presented was ambiguous with regards to the directionality of Rab11a and NP trafficking. To address this, we have replaced Figure 4C with a new experiment in Figure 6, where we have used a cell marker to distinguish between the donor and recipient cells. The CellTracker Blue dye is used to stain the cytosol of the uninfected A549 WT cells which act as the donors for Rab11a. We have used infected A549 Rab11a KO cells as our recipients. High resolution STED imaging of the co-cultured cells in Figure 6A shows Rab11a extending from the blue WT cells through TNTs and co-localizing with vRNPs (as visualized by NP) within the KO cell and the TNT. The use of blue marker and high resolution in this experiment allows us to establish the directionality of Rab11A movement from uninfected to infected cells. Quantitative analysis shown in Figure 6B shows that there is an appreciable level of Rab11a–NP co-localization within the TNTs that connect these co-cultured cells. Taken together with infectious virion production when A549 Rab11a KO cells are infected with delta-HA virus and co-cultured with the Rab11a-sufficient MDCK-HA cells, the data strongly suggest that TNTs allow bidirectional movement of Rab11a.

4) Based on their data showing low efficiency, it seems unlikely that all 8 segments are routinely transported from cell-cell (at least in this system). Indeed, the authors state in the conclusion that “...indicates that vRNPs may be trafficked individually or as subgroups and not as a constellation of 8 segments”. Thus, do the authors think this is a true driver of virus spread and means to evade immune detection? How do the authors think TNTs play a role in infection in the airway? Is this likely between airway cells and other cell types? More discussion on this topic is warranted to better understand the relevance of these findings during natural infection especially since the cell lines and culture conditions used do not recapitulate the tightly packed, polarized epithelial cells in vivo.

We have expanded our discussion to include these points. The relevant text can be found at lines 331-340 and is reproduced below.

“The human airway is composed of multiple cell types, including polarized epithelial cells in a tightly packed environment (35). It is yet unclear what role TNTs may play in the normal homeostasis of the airway, but TNTs have been demonstrated to mediate the transfer of cargo and organelles in various solid tumors of the lung, including adenocarcinomas and mesotheliomas (31). TNT mediated transfer of organelles and other components including viruses has been observed between heterotypic cells, mostly involving immune cells such as macrophages (36–39). It is therefore likely that IAV infection of the airway epithelium or potentially immune cells can spread via TNTs *in vivo*. The extent to which TNT mediated spread acts in conjunction with particle-based transmission of infection within the host is as yet unexplored.”

5) At line 222, the authors speculate that fewer TNT connections may underlie the fact that fewer reassortant viruses are observed when Rab11a is absent from infected donor cells. This argument could be bolstered by quantitation of these connections, and/or citing the literature on this topic (e.g. DOI: 10.1242/jcs.215889; DOI: 10.1038/cddis.2016.441).

We have included quantitation of TNTs observed in WT and Rab11a KO cells in Figure 3. The relevant text can be found at lines 131-139 and is reproduced below.

“IAV infection increases the number of TNTs formed between cells (21,22). To test whether the loss of Rab11a had an impact on TNT formation, we counted the number of TNTs formed between cells in WT and Rab11a KO cells in the presence and absence of NL09 infection. Consistent with prior work, a significant increase in the number of TNTs formed upon IAV infection was observed in both WT and KO cells (Figure 3). Conversely, we saw no significant difference between WT and KO cells in the number of TNTs formed, either in the context of infection or mock infection (Figure 3). The observation that loss of Rab11a does not significantly impact TNT formation suggests that the reliance of vRNP trafficking through TNTs on Rab11a arises through the observed interaction between these components.”

We have also amended our conclusions based on this new information at line 260-262, and is reproduced below.

“Since Rab11a knock out does not impact the number of TNTs formed (Figure 3), this relative lack of reassortants suggests that vRNP transport through TNTs is less efficient when Rab11a is absent from donor cells.”

6) If mutation of either in Rab11a or PB2 to prevent the direct interaction is possible, it may provide a means to decouple the role of Rab11a in TNT formation (indirect) vs. RNP trafficking (direct). At the moment, it seems assumed that Rab11a is responsible for RNP trafficking through the TNTs based on a similar role for Rab11a in bringing RNPs to the site of assembly and the images in Fig 1 (see point #1 above)).

The reviewers make an excellent suggestion for the use of functional mutants. The exact regions of PB2-Rab11a binding are not known, so generation of a specific PB2 mutant that would decouple the interaction is not feasible currently. However, our new data in Figure 3 shows that knock out of Rab11a does not reduce TNT formation, indicating that its role in the spread of IAV infection is not occurring at this level. Conversely, NP within TNTs shows a very high frequency of co-localization with Rab11A, while NP is not seen within TNTs in the context of Rab11A knock out (Figure 2B). Together, the data strongly suggest that the interaction with Rab11a is responsible for vRNP transport via TNTs.

Reviewer #2: Additional Experiments

1. Examine cell-to-cell spread through TNTs in differentiated airways cells at an air liquid interface- The studies presented are restricted to A549 cells sparsely seeded on the coverslip to create the extended filaments between the cells- However, in the airway cells are densely packed together with TNTs are found through tight junctions between cells; thus, a physiologically relevant system should be used to complement the studies presented.

We thank the reviewer for the excellent suggestion of using a more biologically relevant system for analyzing TNT mediated spread. However, there are several technical difficulties in optimizing imaging for differentiated cells at an air liquid interface, which is currently not feasible in our present system. The imaging experiments done on MDCK and A549 cells are performed on sparsely seeded cells to better depict and visualize the TNTs. All other experiments, including the foci formation (Figure 4) and the infectious virus assays from co-cultures (Figure 5) were all performed in densely packed monolayer cultures. Although we agree with the reviewer that a 3D model of TNT spread would be more informative to better reflect *in vivo* conditions, we feel that the time constraints and technical challenges are limiting at present.

2. Image TNTs and the cargo (NP and vRNP) within them using super-resolution or electron microscopy to confirm that they are within the tube and not traveling along the outside of the plasma membrane.

We have included high resolution STED images in Figures 2 and 7 to show the presence of NP and Rab11a within the TNTs. We have also included statistical analyses to show co-localization efficiency of Rab11a and NP within TNTs in Figure 2B and 7B. Please refer to the response to Reviewer 1, point 1 for more details.

3. Examine the role of microtubules on HA-independent cell-to-cell spread. The authors focus on the use of F-actin in TNTs and use Cytochalsin D (actin depolymerization drug) to examine cell-to-cell spread. However, the authors should include a microtubule depolymerization treatment as a control to ensure that the impact on cell-to-cell spread is dependent upon actin and not just on the disruption of cytoskeletal trafficking.

We have included data from Nocodazole treatment of our co-culture experiments in Figure 5D, which shows no detrimental effect of microtubule depolymerization on HA-independent cell-cell spread. The relevant text can be found at lines 211-215 and is quoted below:

“To test if microtubules play a role in TNT mediated transport of IAV genomes, we performed the co-culture assay as described above with vehicle treated and 30 µM Nocodazole, which depolymerizes microtubules. We detected infectious virus in both control and nocodazole treated cells (Figure 5D), indicating that microtubules most likely did not play a role in the trafficking of vRNPs through TNTs.”

**Part II – Other Major Concerns**

1. Details on image acquisition and analysis are missing. It is unclear if authors used computational or manual image analysis pipelines. What were the thresholds used for the analysis etc. It is difficult to interpret the data without these details-

We thank the reviewer for highlighting this issue. We have extended the methods section to include details of image acquisition and analysis.

2. The authors ignore many caveats to the experimental setup and alternative entry pathways, such as:
a. Rab11a has a multitude of roles in intercellular membrane trafficking of cellular and viral components. Presumably, the canonical pathway of IAV assembly would have been disrupted in the knockout system many steps upstream of vRNPs reaching the TNTs.

We have shown in our recent publication (Han et al. 2021), that Rab11a KO impacts but does not abrogate the canonical assembly pathway. We have included this point in the discussion at lines 299-302 and is quoted below:

“Although Rab11a has been shown to be important for trafficking and efficient assembly of IAV genomes (2,8,12), we have shown that the loss of Rab11a does not completely abrogate genome assembly and viral particle release, indicating that there may exist an alternative pathway for canonical assembly of virions (12).”

b. Depolymerization of actin drastically impacts cell morphology that could impact trafficking of vRNP.

We have included data from Cytochalasin D treatment of NL09 WT infected cells (Figure 5C) and show that actin depolymerization has no impact on vRNP trafficking and assembly. The relevant text can be found at lines 205-210 and is quoted below:

“To assess if actin depolymerization was impairing vRNP transport upstream of TNT transfer, we tested the effect of Cytochalasin D on conventional virus production in the context of multicycle replication. No difference in viral yield from vehicle treated or Cytochalasin D treated cells was detected (Figure 5C). Taken together, these data indicate that actin depolymerization specifically abrogates TNT mediated transfer of viral genomes but has no impact on conventional viral assembly.”

c. There may be other strategies of cell-to-cell spread that are HA-independent in addition to TNTs.

We have extended our discussion to include this point. The relevant text can be found at lines 345-349 and is quoted below:

“It has been recently shown that cell-cell spread of H5N1 influenza viruses can occur via trogocytosis in which there is an exchange of the plasma membrane and its associated molecules between conjugated cells (41). This mechanism is also actin dependent and is potentially another route of HA-independent direct cell-cell spread of infection.”

d. The experiments leading to the conclusion of bidirectional Rab11A should be done with live cell imaging since in the current model the Rab11A-mCherry cells could be infected with virus produced in the Rab11A KO cells.

We appreciate the reviewer’s comment and have addressed the ambiguity highlighted with additional experimentation. Please refer to the response to Reviewer 1, point 3.

3. In addition, the viral titers in Fig 4C, suggest to this reviewer that Rab11A is unnecessary for influenza assembly- It is surprising that the authors conclude that viral replication between the WT and KO cells was significantly different when less than a 0.5 log10 decrease was observed. The authors need to resolve why Rab11A would be necessary for vRNP TNT spread and endocytic-based assembly pathways, but not dramatically impact viral titers.

We have shown in our recent publication (Han et al. 2021), that Rab11a KO impacts the canonical assembly pathway, but does not abrogate assembly completely. Our analysis of log transformed data of the viral titers indicates a significant difference between the titers obtained from WT and KO cells (Figure 5E); this modest difference is not important to support the conclusions drawn, however. The data presented here are the titers obtained from co-culture of infected WT or Rab11a KO cells with MDCK WSN HA cells which express Rab11A. As a whole, the system is therefore Rab11A positive. As we show in Figure 7, Rab11a can move in a bidirectional manner within TNTs and grab vRNPs from infected cells, even when the infected cells lack Rab11a.

Reviewer #3: Figure 1. In addition to representative images, quantitative data should be provided to convincingly support the authors' conclusions: how many TNTs were observed in each condition? what were the proportions of NP+ Rab11+, NP+ Rab11- and NP-Rab11+ TNTs?

We thank the reviewer for this suggestion. We have included quantitation of the proportion of NP+ Rab11+, NP+ Rab11- and NP-Rab11+ TNTs in Figure 2C. The relevant text can be found at lines 124-129 and is quoted below:

“To evaluate the frequency with which Rab11a accesses TNTs, we quantified the number of TNTs that were NP+Rab11a+, NP+Rab11a- and NP-Rab11a+ in uninfected and NL09 infected A549 WT cells. Rab11a was routinely detected within the TNTs of uninfected cells, indicating that Rab11a+ recycling endosomes are trafficked within TNTs under normal conditions (Figure 2C). In infected cells, a majority of TNTs carried NP+Rab11a+ puncta, indicating that transport of vRNPs into TNTs is a common feature of the viral life cycle (Figure 2C).”

We have also quantified the number of TNTs observed in WT and Rab11a KO cells under infected and uninfected conditions (Figure 3). The relevant text can be found at lines 131-139 and is quoted below:

“IAV infection increases the number of TNTs formed between cells (21,22). To test whether the loss of Rab11a had an impact on TNT formation, we counted the number of TNTs formed between cells in WT and Rab11a KO cells in the presence and absence of NL09 infection. Consistent with prior work, a significant increase in the number of TNTs formed upon IAV infection was observed in both WT and KO cells (Figure 3). Conversely, we saw no significant difference between WT and KO cells in the number of TNTs formed, either in the context of infection or mock infection (Figure 3). The observation that loss of Rab11a does not significantly impact TNT formation suggests that the reliance of vRNP trafficking through TNTs on Rab11a arises through the observed interaction between these components.”

From a mechanistic perspective it would also be of interest to clarify the following points: in mock-infected cells, were TNTs positive for Rab11 staining, i.e. are Rab11-positive vesicles naturally trafficking through TNTs? Was the average number of TNTs per cell enhanced upon viral infection as described previously by Roberts et al (ref # xx). Was the average number of TNTs per cell modified by Rab11 KO?

Please refer to the response above.

Finally, it would be preferable to state that infected cells show TNTs that are positive for both Rab11 and NP staining, as the imaging resolution is not sufficient to demonstrate colocalization of the two proteins, and therefore does not allow to conclude that “vRNPs associate with Rab11a” within TNTs (line 95), although this association is likely.

We have included co-localization analysis of high-resolution STED images in Figure 2B to demonstrate that vRNPs are associated with Rab11a within TNTs. Please refer to the response to Reviewer 1, point 1.

Figure 4. As high MOIs are used for the initial infection and low infectious titers (~10e3-10e4 PFU/ml) are detected in the supernatant of the coculture, it is important to check that there is no residual infectivity upon acid wash. The percentages of Venus-positive and Scarlet-positive cells in the coculture should be provided to allow a thorough interpretation of the data showed in Figure 4 and Figure 6.

We have included titers post-acid wash (0h) to show no residual infectivity (Figure 5). Unfortunately, the mScarlet fluorophore is not visible under epifluorescence, so quantitation of positive cells is not feasible.

Figure 5. Figure 5 does not convincingly demonstrate that Rab11 transfer from non-infected cells to infected cells could account for TNT-dependent cell-to-cell spread in the experiment shown in Figure 4B. In DMSO cells, could NP+ mCherryRab11+ TNTs be observed? if yes, was the presence of NP in the TNTs strictly dependent upon the presence of mCherry-Rab11? if no, can NP+ mCherryRab11+ TNTs be observed at later time-points?

We appreciate the reviewer’s comment and have addressed the ambiguity highlighted with additional experimentation. Please refer to the response to Reviewer 1, point 3.

To further confirm the author’s hypothesis of Rab11 transfer from non-infected cells to infected cells, the experiment shown in Figure 4B should be performed using MDCK-WSN-HA cells depleted in Rab11a, eg pre-treated with siRNAs targeting Rab11a.

We agree that depletion of Rab11a in MDCK WSN HA cells would be a valuable approach; however, the transfection efficacy of siRNAs in MDCK cells is very low (about 10%). This technical limitation makes the approach unfeasible.

**Part III – Minor Issues: Editorial and Data Presentation Modifications**

Reviewer #1: 1) Figure 6 would benefit from additional labeling within the figure itself.

Figure 6 has been replaced with Figure 7 and we have endeavored to include clear labels within the figure.

2) Line 58 should be either “comprises eight RNA” or “is composed of”

Change has been made.

3) Line 97 seems to be missing a reference after the first sentence of the results “Upon nuclear exit...”

Change has been made.

4) Line 144 should read “shown in Figure 3”

Change has been made.

Reviewer #2: 1. All images presented in this manuscript are small include a wide field of view. They would benefit from zoomed-in insets of regions of interests, highlighted interactions within the tunneling nanotubes, and a scale bar needs to be added.

We have included magnified insets of the high-resolution STED images and highlighted interactions of Rab11a and NP within the TNTs in Figures 2A and 7A. Scale bars have also been added to the images.

2. Line 144 – missing “in” before “figure 3, this is...”.

Change has been made.

3. Fig. 6 lacks the “A” and “B” subpanel labels mentioned in the text.

Thank you for catching this error. Figure 6 has been replaced with Figure 7.

Reviewer #3: Figure 2 and 3. The effects of cytochalasin or Rab11-KO are quite striking, in that they suppress the increase in foci number over time. However, the authors should show a baseline i.e. the same counts at an earlier time point such as 4-6 hpi, to demonstrate that the ~50 foci detected in the presence of cytochalasin or Rab11-KO were most likely formed during the initial infection round. It would also be interesting to show analysis of the same experiments with a higher threshold of ≥3 cells instead of ≥ 2 cells per foci.

We appreciate the reviewers’ point about determining the baseline number of foci formed at inoculation. However, the mVenus fluorescence used as a readout for focus counts is only detectable at 16h post infection (and later). Therefore, earlier time points cannot be analyzed in this experiment. The higher threshold of ≥3 cells yields very few foci that can be counted, hence the threshold of ≥ 2 cells per focus has been used for this analysis.

Figure legends. Indications regarding MOI and time-point should be provided in the main text and/or legend figure for each experiment. Line 534 and line 540, the number of independent experiments should be indicated. Line 549: whether the three replicates are technical or biological replicates should be clarified.

MOIs used for infections and replicates have been indicated in the figure legends.

In the introduction, line 60-68. When referring to the role of Rab11 in the trafficking of newly synthesized vRNPs, the recent review by M Amorim (PMID 30687703) should be cited in addition to ref #2. Recent mechanistic insights into the transport of Rab11-vRNP complexes provided by the studies of Alenquer et al (PMID 30967547) and de Castro Martin et al (PMID 29123131) should be cited in addition to ref #5-8.

We thank the reviewer for highlighting these relevant prior studies. The references have been added (References 3, 10-11).

Line 96. Appropriate references (PMID 21307188 and 23063830) should be cited to support the notion that vRNPs bind to Rab11 via PB2.

These references have been added (References 8,24)

Line 248. The manuscript provides no evidence that loss of Rab11a leads to a more “dispersed NP localisation within the cytoplasm”. The sentence should be rephrased.

We have included high resolution STED images to show NP-Rab11a co-localization within TNTs in Figure 2A. We have also included statistical analyses to show co-localization efficiency of Rab11a and NP within TNTs in Figure 2B. These results are described in the modified text at lines 125-136, as follows:

We used super resolution Stimulated Emission Depletion (STED) microscopy to analyze the association of Rab11a and vRNPs within TNTs in more detail. STED microscopy overcomes the diffraction resolution limit of confocal microscopy and allows for imaging with up to 30 nm resolution (24,25). A549 WT or Rab11a KO cells were infected with NL09 viruses and stained for NP, Rab11a and F-Actin. Using 3 color STED imaging of TNTs, we observed punctate Rab11a and NP staining in close proximity within the TNTs in WT cells. In the case of the Rab11a KO cells, the NP staining was relatively diffuse in the cytoplasm and could not be observed within the TNTs (Figure 2A). Co-localization of Rab11a and NP within TNTs was analyzed quantitatively for both WT and KO cells. The observed colocalization co-efficient of 0.9 in the WT cells indicates that the majority of the NP puncta within TNTs coincide with Rab11a puncta (Figure 2B). These data indicate that Rab11a and NP are indeed interacting with each other within TNTs and are likely trafficked as a complex.

Line 51. The manuscript provides no evidence that “complexes of Rab11a and viral components can be trafficked?” across tunelling nanotubes. The sentence should be rephrased.

We have included co-localization analysis of high-resolution STED images in Figure 2B to demonstrate that vRNPs are associated with Rab11a within TNTs.
