# Supplementary material for: Rab11a mediates cell-cell spread and reassortment of influenza A virus genomes via tunneling nanotubes
Source: PLoS Pathog. 2021 Sep 2;17(9):e1009321. doi: 10.1371/journal.ppat.1009321 (PMC8443049; doi:10.1371/journal.ppat.1009321)
Supplement: Attachment — Submitted filename: Response to Reviewer comments-2.docx [file ppat.1009321.s006.docx]

Dear Dr. Dittmann,

We are happy to submit a revised version of our manuscript entitled “Rab11a mediates cell-cell spread and reassortment of influenza A virus genomes via tunneling nanotubes." (PPATHOGENS-D-21-00131R1). We have reproduced the reviewers' comments on the following pages and detailed our responses underneath in blue font. The modifications made include the addition of clarifications within the text. The reviewers provided insightful comments and we would like to thank them for improving the quality of this work.

Sincerely,

Ketaki Ganti and Anice Lowen

**Part III – Minor Issues: Editorial and Data Presentation Modifications**
Please use this section for editorial suggestions as well as relatively minor modifications of existing data that would enhance clarity.

Reviewer #1: -The cells in Figure 2A should be labeled as Rab11a KO in the figure for clarity
-Line 157 – I believe the authors mean Venus-positive cells as these are HA deleted viruses here.

We thank the reviewer for catching this error and we have made the corrections.

Reviewer #2: 1. Since tight junctions could function similarly to TNT in differentiated airways and other viruses (hMPV and measles viruses – see work by Dutch Lab and Cattaneo Lab) have been shown to travel cell-cell through these sort of connections it is feasible IAV would as well. Inclusion of this point in paragraph starting on line 334 would be nice.

We agree with the reviewers point and appreciate the suggestion. We have included a statement at lines 372-375, reproduced below:

“Direct cell-cell spread of human metapneumovirus occurs via reorganization of the actin cytoskeleton (42), while measles virus infection of neighboring cells can occur through tight junctions (43,44); whether IAV exploits these two routes of spread remains unclear. “

2. STED imaging comments:
a. Was there any correction for chromatic aberration?

Yes. We have included the details in the methodology at lines 483-485 and is reproduced below:

“The microscope is a custom design with a built in Olympus IX3-ZDC-12 z-drift compensation unit for steady confocal/STED imaging to correct for chromatic aberration.”

b. What was considered in “close proximity”? An image resolution of 30 nm can provide a more precise quantification for colocalization between Rab11a and NP is possible in an unbiases computational manner (rather than manual counting).’

We agree that a resolution of 30nm would be the most precise in quantifying colocalization. However, due to the fluorophores that we have used in this study, the maximum resolution achieved was 41.67nm. Co-localization efficiency of NP and Rab11a was calculated using the using the JaCoP plugin in FiJi.

c. Was a 30nm resolution confirmed for your specific images and setup? If not – please provide the limit of resolution.

We achieved a resolution of 41.67nm and this is described in lines 130-132 and reproduced below:

“A resolution of 41.67 nm for the Rab11a-NP staining was achieved, giving a high degree of confidence in the co-localization quantitation. Of note, Rab11a was previously determined to interact with vRNPs via PB2 (24) and not with NP, the target of our vRNP staining.”

d. In Fig 2 and 6 the resolution of the high resolution images is still pixelated when zoomed in. Please consider making the zoomed images larger so that the power of the STED images can be revealed.

The images in Figures 2 and 6 have been modified to better depict the zoomed insets.

3. Fig 1 – no scale bars is included. The legend of the cells is inaccurate. The resolution and image size makes it difficult to assess colocalization.

Figure 1 has been modified to include scale bars. The legend now reflects the figure accurately. We assess colocalization in Figures 2 and 6.

4. Fig 2 – what is the time point of infection? Was the mander’s colocalization coefficient used only on TNT region of interest? Please include that detail in the methods. Also, was the coefficient derived from a single slice or an 3D volume of the cell? Please include these details in the methods.

The time point of infection is noted in the methods at line 4. The Mander’s coefficient was only applied to the TNT regions of interest and only on the single slice of the 2D image. The detail has been added to the methods at lines 504-505 and is reproduced below:

“The quantification of co-localization was limited to the TNT regions of interest. All images were acquired in 2D and quantification was performed on the single slice.”

5. Fig 6 – include the number of independent replicates for this study. What was the time post infection. The Rab11a signal is interested since it is present in the cytoplasm of the KO cell as well. Please comment on whether the same staining procedures, imaging parameters and pixel intensities are consistent between this data set and the STED images in Fig 2 of Rab11a KO cells.

The legend has been modified to include the number of replicates at lines 780-781. The time post infection is noted in the methods at line 480. All images were acquired using the same staining procedures and imaging parameters. We have included this information in the methods at lines 496-497.

6. Please note that STED images do not indicate direct interaction. The examination of PB2 and Rab11a direct interaction is most convincingly shown in Avilov et al – using bi-fluorescence complementation assay with split GFP on PB2 and Rab11a. Consider modifying line 122.

We have modified the statement and included the reference (Reference 24) at lines 130-132 and is reproduced below:

“A resolution of 41.67 nm for the Rab11a-NP staining was achieved, giving a high degree of confidence in the co-localization quantitation. Of note, Rab11a was previously determined to interact with vRNPs via PB2 (24) and not with NP, the target of our vRNP staining.”

Reviewer #3: In the absence of live imaging data, the authors’ claim that they find evidence for bidirectional movement of Rab11 through TNTs (e.g line 226, lines 276-277) should be attenuated. The experiment shown in Figure 6 does not prove bi-directionality. The only argument for bidirectionality is the production of viral progeny when A549-Rab11KO cells are co-cultured with MDCK-HA cells (Figure 5E), however it is a very indirect argument. Therefore bidirectionality may be referred to as likely, but not certain.

The title of the relevant results section was modified to “***TNTs likely allow bidirectional shuttling of Rab11a between cells”***

We have also modified our conclusions at lines 259-260 and this is reproduced below:

“Our data suggest that TNTs formed in the context of IAV infection are most likely open ended and bi-directional.”

Line 221: it should be clarified “with a marginal difference in titers at 48 and 72 hpi ”, as the difference in titers between A549-WT and A549-Rab11KO cells is not marginal at 24 hpi.

We have made the modification at line 240.

Figure 2: the inclusion of STED imaging and quantification data represent a significant improvement. However the confocal and STED images shown in Figure 2A are very pixelated, and higher quality images should be provided (the same comment applies to Figure 6). The labeling of the right panel of Figure 2A should state « Rab11a-KO ».

The images in Figures 2 and 6 have been modified to better depict the zoomed insets.

In Figure 2B, does it really make sense to quantify the co-localisation of NP with Rab11 in Rab11-KO cells?

We wanted to depict conclusively that NP is not found within TNTs in the absence of Rab11a which is why we have included the quantification of co-localization in the Rab11a KO cells.

In the introduction, lines 65-71: the notion that an intact microtubule network is important for the transport of vRNP should be tempered, as several pulbications have reported only a modest decrease of the production of infectious viruses upon treatment with nocodazole. The authors themselves, in Figure 5D of the present manuscript, show no effect (or even a positive effect) of nocodazole treatment on the productious of infectious viruses.

We have amended our interpretation at lines 65-69 and is reproduced below:

“The intracellular transport of vRNP-Rab11a complexes is thought to occur via the microtubule network with the help of dynein motors (6–11). There are, however, conflicting observations about the impact of microtubule disruption on the viral lifecycle, with results ranging from no detectable effect (6) to an attenuation of viral progeny production (8)."
